# Segregate, Test, Observe and Persevere (STOP): strengthening ex situ breeding programs for biodiversity in zoos amid highly pathogenic avian influenza threats - a case approach

**DOI:** 10.3389/fvets.2026.1708354

**Published:** 2026-03-06

**Authors:** Anne Günther, Marco Roller, Lukas Reese, Ulrike Foldenauer, Judith Tyczka, Anne Pohlmann, Martin Beer, Dominik Fischer, Timm Harder

**Affiliations:** 1 Institute of Diagnostic Virology, Friedrich-Loeffler-Institut, Greifswald, Germany; 2 Zoological Garden Karlsruhe, Karlsruhe, Germany; 3 Food Monitoring and Veterinary Department, Public Order Office of the City of Karlsruhe, Karlsruhe, Germany; 4 State Institute for Chemical Analysis and Veterinary Diagnostics Karlsruhe (CVUA), Karlsruhe, Germany; 5 Zoo Wuppertal, Wuppertal, Germany

**Keywords:** wild birds, ex-situ breeding program, zoological institution, wildlife-zoo interface, disease management

## Abstract

Zoos with avian populations are vulnerable to incursions of the high pathogenicity avian influenza virus (HPAIV) H5Nx due to the free-range husbandry, bird population density and shared open water areas between zoo birds and wild waterfowl. An outbreak of HPAIV H5N1, clade 2.3.4.4b, genotype EA AB, at the Zoological Garden Karlsruhe, Germany, in 2022, was managed applying legal restriction measures but exceptionally exempting culling orders for RT-qPCR-positive but clinically healthy birds. A critical factor in the zoo’s response was the implementation of a segregation concept approved in advance. The entire bird population could be rapidly separated into epidemiological housing units (epUs) cared for by separate staff. A total of 79 birds initially tested RT-qPCR-positive, but only 21 (26.6%) clinically diseased birds had to be euthanized or succumbed. Seroconversion amounted to 94.8% of the remaining 58 birds. Extensive RT-qPCR investigations of 3,634 samples confirmed infections remained confined to three initially infected epUs out of a total of 25. Spread of virus in the infected epUs was limited to 3 weeks after segregation. In the cohort of infected pelicans, surviving individuals remained seropositive with elevated levels of H5-specific antibody titers for the following 2 years suggesting ongoing protection. The described science-based control measures rested on a legally binding yet interpretive statement regarding the pertinent animal health legislation are exemplary for managing an outbreak of high pathogenicity avian influenza (HPAI) in zoos.

## 1 Introduction

For several years, high pathogenicity avian influenza viruses (HPAIV) of the H5Nx subtype, evolving since 1996 from the Goose/Guangdong/1/96 lineage (gs/GD) of Eurasian subtype H5 viruses, have expanded their geographic and host range among wild birds. Annual circulation of HPAIV H5 of clade 2.3.4.4b has been confirmed in Europe (1). This has led to a continuous cycle of mutual transmission of these viruses among and between domestic poultry, other captive birds, and wild birds (2). The resulting persistent risk of HPAIV incursions poses a significant challenge for zoos and analogous avian collections (3). Anseriformes and Pelecaniformes, in particular, represent delicate bird orders regarding husbandry. In terms of animal welfare, those species have to be kept with access to a fresh water source, preferably outdoors. Those sources are preferred in zoos, as they model the natural habitat of the species best and are more appreciated by zoo animals and zoo visitors. However, water sites are known to likewise attract wild birds with similar habitat preferences (e.g., mallards) or species foraging in the left overs of provided animal food (e.g., herons, storks, gulls, and crows). These tight wildlife-zoo animal interfaces lead to a conflict of interest, whereby more natural or integrated conditions of husbandry may result in increased exposure to wild birds as natural reservoirs for pathogens. In this respect, natural husbandry of zoo birds suffers the same limits as free-range holdings of poultry, where efficient biosecurity measures would often be diametrically opposed to the intentions of the rearing system. Wild water birds, such as gray herons, mallards and geese species, represent natural reservoir species for HPAIV, and additionally act as bridging hosts between the zoo areal and other wild bird-populated sites of the region (e.g., city parks and wetlands).

In recent years, the enzootic presence of gs/GD-like HPAIV in wild birds has increased the risk of HPAIV incursions into zoos. Outbreaks have been shown to cause elevated mortality among many of the exposed bird species (4). Suspected cases of HPAI require prompt notification at the veterinary authority who initiates official testing and restriction procedures. In HPAIV-positive premises, the provisions of the Animal Health Law (AHL) for the control of animal diseases (5) apply within the European Union (EU). In the context of poultry farming, this requires the obligatory culling of the affected flock (6, 7). Exemptions from this culling obligation may be granted by the competent authority in the event of rare breeds of poultry or in zoological collections, provided that certain conditions are met which aim to hamper further spread of virus from the affected premises (7). Exemptions in zoos are possible since they can be defined as “confined establishments” within the provisions of the AHL where the risk of horizontal spread of the pathogen to other zoos, poultry flocks or the environment can be assumed to be negligible.

There is an overall lack of published experience with the eradication of HPAIV from affected zoos that comply with the conservation of valuable and endangered species. Authorities tended to view zoos as a single epidemiological unit (epU) and applied measures in a generalized manner. However, the concept of epUs was considered to some extent when tackling more recent outbreaks, for example in culling activities (4). Preventive and eradicative measures put considerable strain on the zoo’s human and financial resources, and the overall animal welfare. In this use case approach, we conducted longitudinal virological and serological studies in the Zoological Garden Karlsruhe, Germany, following an HPAI outbreak that had been countered by a particularly rapid fragmentation into several epUs and accompanied by comprehensive surveillance and allowed for recovery of infected species.

Our intensive monitoring revealed the routes of entry and spread within the zoo and aided in tracking H5-specific immunity in some bird species. We specify steps toward minimizing culling of clinically inconspicuous animals.

## 2 Materials and methods

### 2.1 Description of clinical monitoring and sampling design

**Figure 1:**
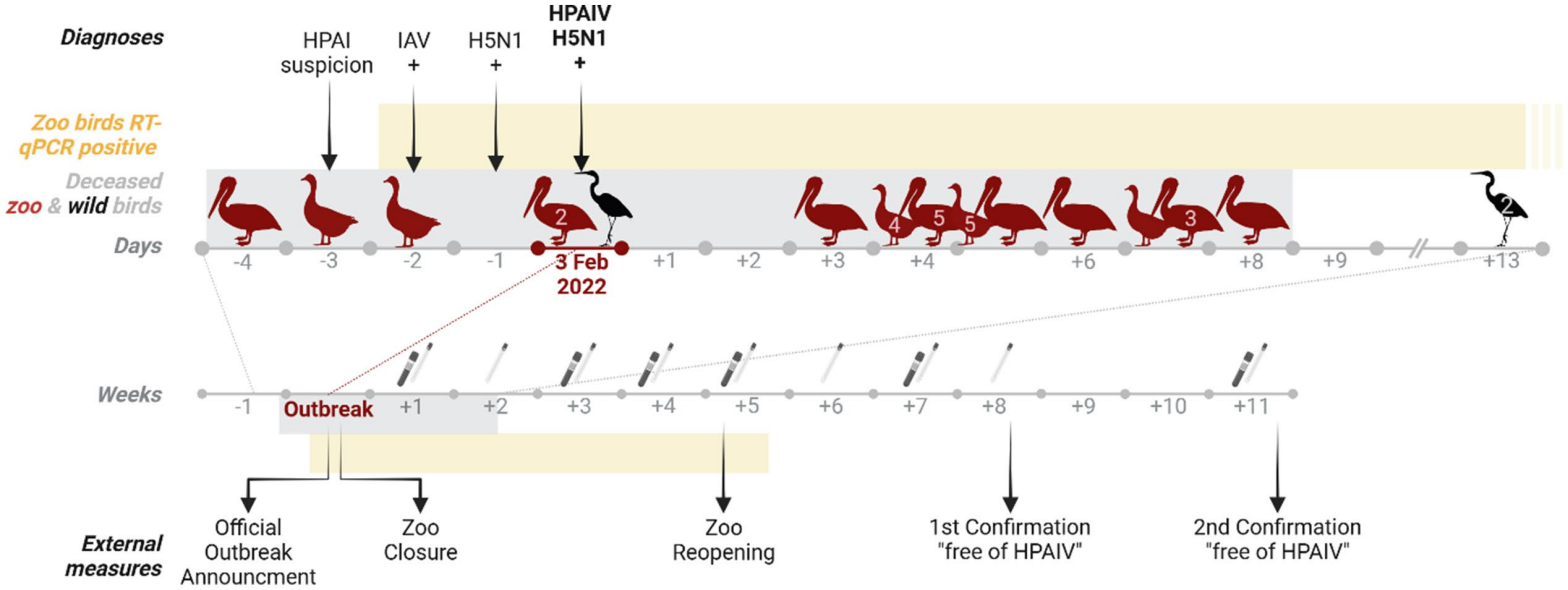
Timeline of events regarding an HPAIV incursion into the Zoological Garden Karlsruhe. The time points (arrows) at which diagnoses were made and external measures were implemented are listed, as well as the time periods during which zoo birds were tested positive for HPAIV via RT-qPCR (yellow block) and HPAIV positive individuals were found deceased or euthanized (gray block; red shapes: zoo bird species; black shapes: wild bird species) during the outbreak. The numbers inside the bird shapes indicate the total number of deceased individuals per species at each time point. Numbers below the days or weeks axis indicate the number of days or weeks before or after confirmation of the outbreak on 3 February 2022, which is regarded as Day 0. The swab icon indicates timepoints when combined or fecal swabs were taken for RT-qPCR and the blood tube icon when blood was taken for serology. Created in BioRender. Günther, A. (10) https://BioRender.com/vcgdz50.

In order to obtain a rapid overview of the overall epidemiological situation, aviaries and outdoor enclosures were grouped to form epidemiological units (epUs). High and low risk epUs were defined according to contact with open ponds in the zoo or contact with confirmed HPAI cases. High risk units were sampled using combined choanal and cloacal swabs, lower-risk units were sampled using fecal swabs only. The clinical monitoring was carried out by specifically trained animal keepers during the daily care of the animals and consisted of a visual examination of the animals with a focus on general health and neurological symptoms. An extended (twice daily) clinical monitoring by veterinarians was followed up on suspicion and combined with the mandatory sampling. The sampling design and procedures were drawn up by the regional State Institute for Chemical Analysis and Veterinary Diagnostics (CVUA) and ordered by the veterinary authorities on basis of provisions from the national Animal Disease Control Manual (8). They included at least weekly sampling of birds of each epU either by combined choanal and cloacal swabs or fecal swabs (see 2.2.). Sampling schemes were based on an infection risk assessment and supervised by official veterinarians. Species exempt from mandatory indoor housing (penguins and ratites) were sampled three times a week. Blood samples were taken repeatedly from infected units during the sampling period (Figure 1).

### 2.2 Sampling procedures and sample storage

Combined swabs of birds in high-risk units had been collected by swabbing the oropharyngeal mucosa near the bird’s choana, followed by using the same swab to sample the cloacal mucosa. The number of fecal swabs per low-risk unit was based on the total number of individuals housed in the respective units: 60 swab samples if there were more than 60 individuals enclosed; the number of swab samples was proportional to the total number of individuals if there were fewer than 60 individuals enclosed. This ensured that the disease could be detected within an epU with at least 95% statistical certainty from a prevalence of 5% onwards, depending on the epU’s population size (9). All swabs were immediately transferred in virus cultivation medium and transported to the CVUA. All zoo birds were adult at the time points of sampling. A few potentially susceptible non-avian species were also sampled in week 2 after the outbreak, including domestic pigs (*Sus scrofa domesticus*), Californian sea lions (*Zalophus californianus*) and polar bears (*Ursus maritimus*) using fecal swabs.

**Figure 2:**
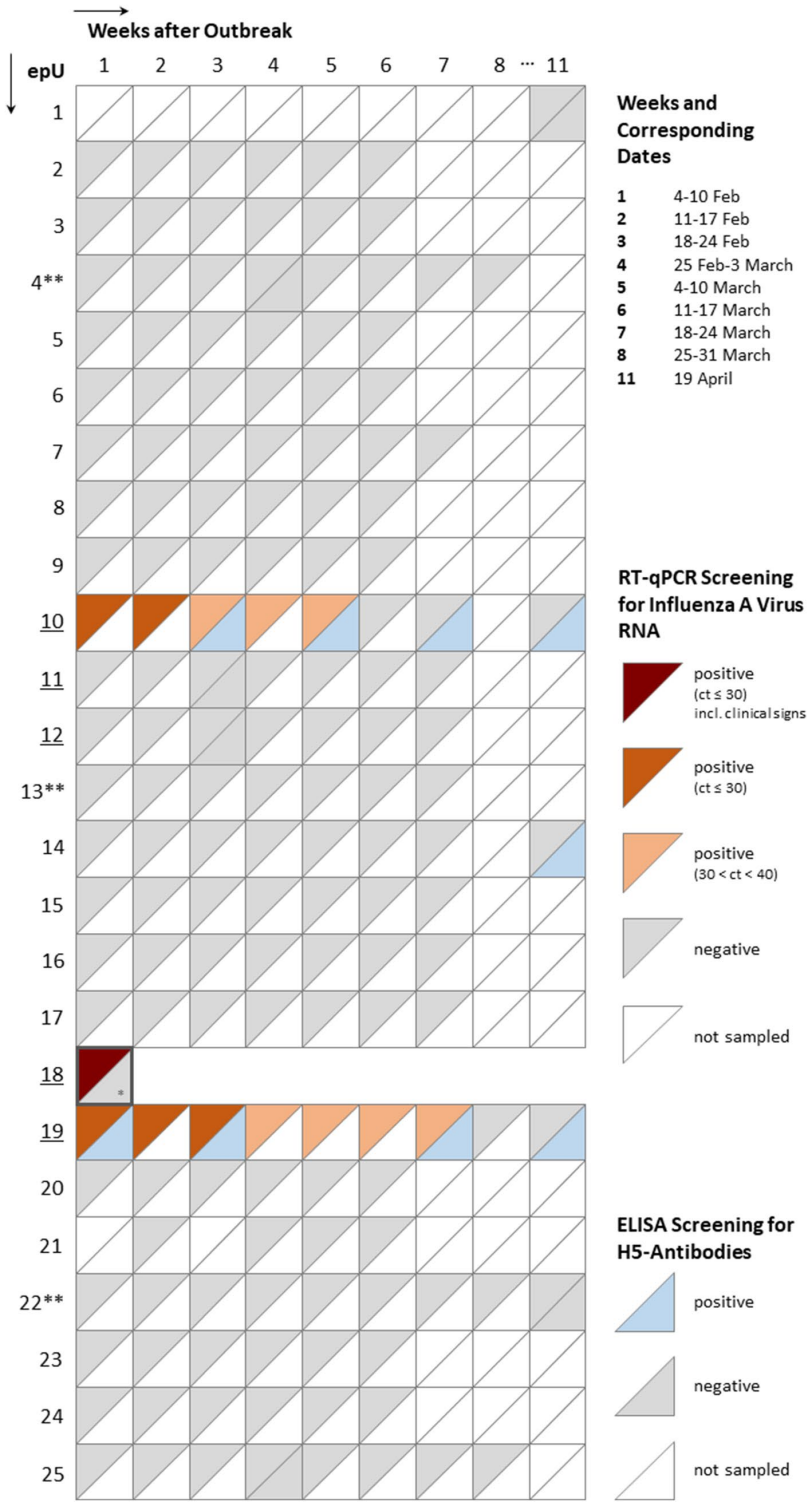
Composite overview of summarized clinical, virological, and serological results of surveillance activities in 25 epidemiological units (epUs) formed after an outbreak of HPAIV in the Zoological Garden Karlsruhe, Karlsruhe, Germany. The columns mirror the time point of sampling. Squares symbolize sampling events in the different epUs. The upper left corner of each square refers to virological screening results with a graded color scale (red) indicating the viral load based on cycle of threshold values (ct). The lower right corners reflect positive (blue) serological screening results for antibodies against subtype H5. Underlined epUs indicate those that were initially classified as high risk epUs. * - Partial sample set due to limited material; ** - for animal welfare reasons, an exemption from indoor housing-order was granted for penguin and ratite species (epUs 4, 13, and 22).

Blood samples were collected from the infected individuals and sporadically also from individual birds in epUs that tested RT-qPCR-negative (e.g., 1; Figure 2). Samples were collected using a 3 mL syringe and a 23G needle from the *Vena metatarsalis plantaris superficialis* in Anseriformes, *V. jugularis* in Gruiformes and Psittaciformes, *V. coccygea dorsalis* in Sphenisciformes and *V. ulnaris* in Struthioniformes. Blood samples were centrifuged for 5 min at 3000 rpm for obtaining serum and stored at −20 °C until further processing at the National reference laboratory for Avian Influenza (NRL AI) at the Friedrich-Loeffler-Institute (FLI), Greifswald, Germany.

### 2.3 Definition of humane endpoint criteria

Contrary to common culling practice, infected animals with clinical signs of avian flu were euthanized only when humane endpoints were reached. Humane endpoint criteria included apathy, stop of intake of feed and water and neurological symptoms such as ataxia, circular movements, tremor and head tilt. Euthanasia was carried out in accordance with good veterinary practice. The animals were euthanised by intravenous or intraperitoneal administration of pentobarbital (400 mg/kg, Narcoren 16 g/100 mL®). Treatment of HPAI-infected animals remained forbidden.

### 2.4 Viral genome detection using RT-qPCR

RNA was extracted from swab sample supernatants using the QIAamp Viral RNA Mini Kit on the QIAcube (QIAGEN, Hilden, Germany) for single samples and MagMAX™ CORE Nucleic Acid Purification Kit on the KingFisher™ Flex Purification System (Life Technologies, Darmstadt, Germany) with 96 Deep-Well Head for sample series. Subsequent testing of the RNA via RT-qPCR with Kylt IVA beta RT-qPCR kit (SAN Group Biotech Germany, Höltinghausen, Germany) aimed to target the matrix gene (M) of influenza A viruses (IAV).

### 2.5 Antibody detection using ELISA and hemagglutinin inhibition assay

All serum samples had been heat-inactivated for 2 h at 56 °C before testing. All available samples were applied in succession to two commercially available ELISA kits. The first ELISA tested for the presence of antibodies against the nucleoprotein (NP) of IAV (ID Screen® Influenza A Antibody Competition, Innovative Diagnostics, Grabels, France) and the second ELISA tested the NP-positive samples for the presence of antibodies against the subtype H5 (ID Screen® Influenza H5 Antibody Competition 3.0 Multi-species, Innovative Diagnostics, Grabels, France). Both analyses were run according to the manufacturer’s instructions. H5 antibody positive samples were tested in a hemagglutinin (HA) inhibition assay (HI) against three avian influenza virus (AIV) antigens (H5N1-A/ck/Scotland/1/59; H5N3-A/Teal/England/7394–2,805/06 and H5N8-A/tk/Italy/7898/14 [clade 2.3.4.4]) as described (10).

### 2.6 Sequencing of AIV

Selected zoo bird swab samples were prepared for sequencing, in addition to available wild bird samples that tested positive in the surroundings of Karlsruhe in winter 2021/2022 (Supplementary Table S1). The MinION-based sequencing approach and the calculation of the maximum likelihood (ML)-tree for the HA segment align with the descriptions provided in Halwe, Cool, Breithaupt, et al. (11). The concatenated coding sequences of all segments were generated and used to determine the genotype as described in Pohlmann and Harder (12). A brief summary of the procedure can be found in the Supplementary Annex. Throughout the manuscript we applied the nomenclature proposed by the European Reference Laboratory (EURL) for Avian Influenza, Padova, Italy. All sequences generated within this study had been uploaded to GenBank and are available under the accession numbers PX528872 - PX528991. The calculation of the ML-tree (Supplementary Figure S1) is based on a Global Initiative on Sharing All Influenza Data (GISAID) data base query, including complete HA segments from animal hosts sampled between October 2021 and March 2022 in Europe (EPI_SET_250826se; 10.55876/gis8.250826se). Median-joining network analyses for alignments of genotype EA AB sequences with a focus on the zoo bird sequences from Karlsruhe and from wild birds in the vicinity of the zoo had been created using the software “POPART” (13).

### 2.7 Visualization

**Figure 3:**
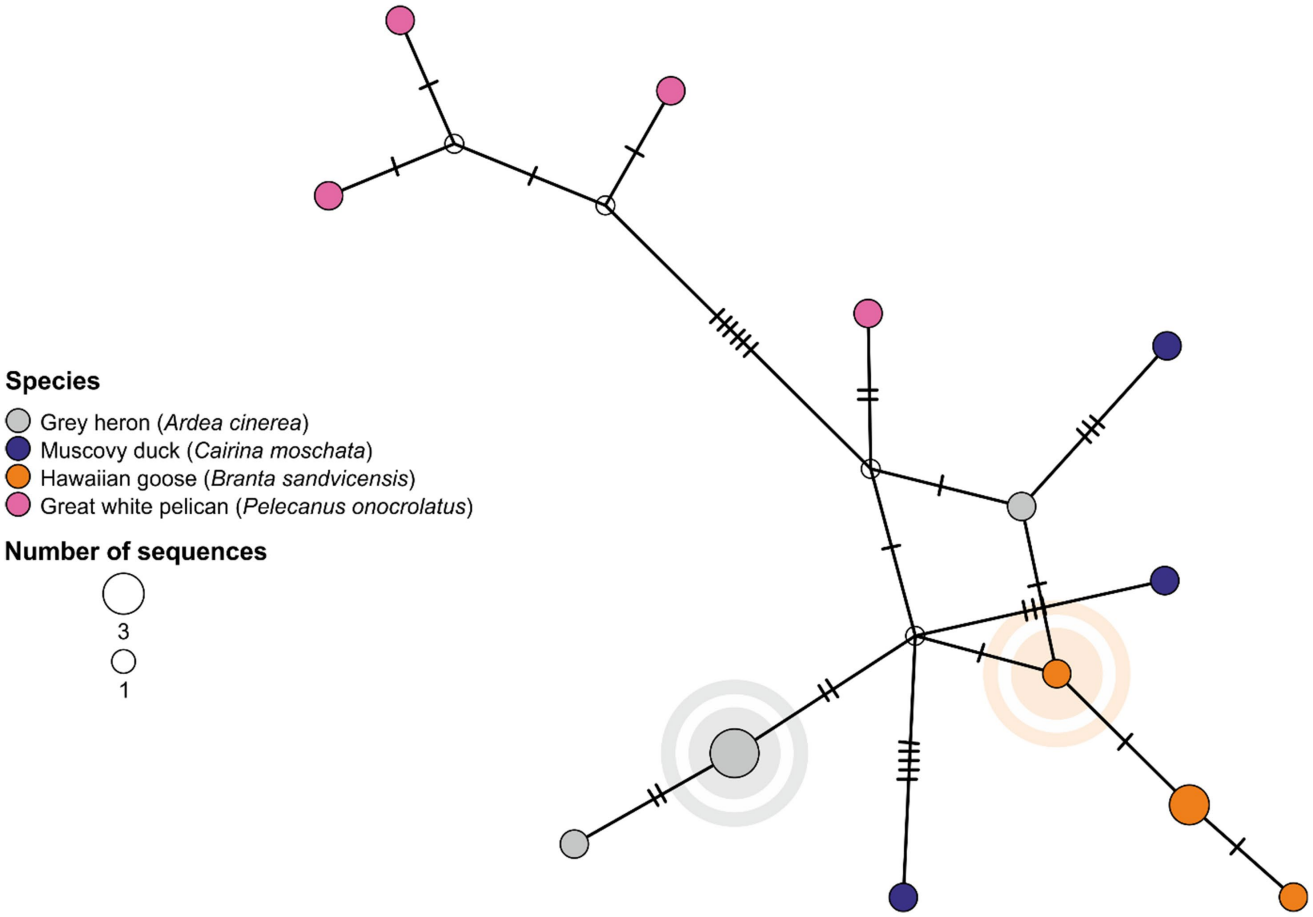
Median joining network of the concatenated coding sequences of all eight influenza A virus genome segments of HPAI viruses H5N1 2.3.4.4b, Eurasian (EA) AB genotype, associated with an outbreak in the Zoological Garden Karlsruhe, Karlsruhe, Germany. The sequences of the wild bird (index) cases take a central position in the network with all sequences of zoo birds being localized at more peripheral sites. Unique sequences are depicted in uniformly sized circles colored by species. Enlarged circles represent a minimum of two identical sequences. Branches connect the phylogenetically related sequences as based on nucleotide identity with marks indicating the number of mutations. Calculated ancestral/intermediate sequences remain uncolored. The network indicates incursion of EA AB virus from wild birds, likely gray herons, into the zoo and further spread among birds having access to open water bodies in the zoo. Index cases in wild birds (gray heron) and zoo birds (Hawaiian goose) are highlighted.

Shapes of avian species were downloaded from Phylopic.org, all of them published under CC0 1.0 Universal Public Domain Dedication license. Figure 2 was created via Microsoft Powerpoint, while Figure 3 was obtained by “POPART” and was edited in CorelDRAW 2017. Graphs depicting data points are created using GraphPad Prism version 10.5.0 (673) for MacOS, GraphPad Software, Boston, Massachusetts USA.

## 3 Results

### 3.1 HPAI H5 case report

#### 3.1.1 Background situation: ongoing HPAI enzootic in Southwest Germany, 2022

Between July 2021 and June 2022, a total of 179 wild birds were tested for presence of IAV RNA for the administrative district Karlsruhe in the context of a nationwide passive wild bird surveillance program. A total of 11 individuals of five different species tested IAV positive by RT-qPCR, exclusively in the period between late January and late April 2022 (Supplementary Table S1).

The first HPAIV H5 RNA positive case in January 2022 was a gray heron (*Ardea cinerea*), showing weakness and neurological signs. It was found alive in a pond located in a park of the city of Karlsruhe (about 2 km linear distance to the zoo), but was euthanized due to the severity of clinical signs. RT-qPCR assays confirmed HPAI virus subtype H5N1, clade 2.3.4.4b. While nine further wild bird samples also revealed subtype H5N1, subtype H5N2 was identified in two samples retrieved from two swans (*Cygnus* sp.; Supplementary Table S1; all sequencing results see 3.2.2).

#### 3.1.2 HPAI H5 outbreak in the zoological garden Karlsruhe: clinical suspicion, laboratory confirmation, pathological examination and initial actions

A comprehensive timeline of the outbreak in the zoo is depicted in Figure 1. At the end of January, one Great White pelican (*Pelecanus onocrotalus*), kept in one of the ponds beneath a gray heron colony, showed sudden apathy, hemoconcentration and leukocytosis (Day −4) and died 1 day after initial treatment. On the following day, one Hawaiian goose (*Branta sandvicensis*) was euthanized with mild neurological signs (Day −3), leading to notification of an initial suspicion of HPAI (zoo index cases, Day −1). A second Hawaiian goose was found dead a day later, when an IAV infection of both initial cases was obtained by RT-qPCR (Day −2).

The necropsy of the pelican showed a generalized reddening of muscles and organ tissues. In addition, multifocal petechiae were present in the coronary fat and the bird showed intestinal contents of soft consistency with a reddish discoloration (suspected hemorrhagic enteritis). At necropsy, both Hawaiian geese showed reddened, compacted lungs, hyperemia of the liver, disseminated miliary gray foci in the pancreas and a high-grade vascular injection of the leptomeninx.

Four days after the first death on February 3, 2022, HPAIV H5N1 was officially confirmed at the NRL AI at the FLI (Day 0). Already 1 day prior to the official HPAIV-confirmation, almost all birds were segregated in previously defined epUs (see paragraph 4.1.3). At this stage, several veterinary colleagues from other zoos, specialized veterinary institutes and universities were consulted in order to optimize all further steps.

A total of four HPAIV H5N1-positive gray herons, three found in the zoo, one further found in its vicinity, are suspected to have accessed a gray heron colony located in the trees directly above some of the zoo ponds. This was suspected as an epicenter of the zoo outbreak. However, due to species conservation legislation, no sufficient measures were permitted to reduce or eradicate the wild bird population within the zoo, in order to reduce direct or indirect contacts with zoo birds.

#### 3.1.3 Application of legal regulations and implementation of adapted control measures

On basis of Article 13 (2) (a-d) of the Commission Delegated Regulation (EU) 2020/687, the competent authority agreed to issue an exemption from culling (see Supplementary Annex). To prevent further transmission of a category A disease and to provide the necessary biosecurity measures the zoo was closed to the public. Animal movements in and out of the zoo were stopped. Measures to effectively prevent the spread of the disease through visitor traffic when reopening for visitors, in order to fulfill essential tasks in accordance with EU Directive 1999/22/EC and for economic reasons, included closure and cordoning of all areas where birds were kept. For visitors, disinfection mats at the entrances and exits, installation of signs with the inscription ‘Avian influenza - unauthorized access prohibited’ in restricted areas and a comprehensive concept for informing visitors about the current epidemiological situation were established.

Derogation from culling comprised infected but clinically inconspicuous birds, while adhering to strict biosecurity measures and embarking on a continuous reassessment and surveillance scheme.

A total of 25 epUs were formed according to a thorough risk assessment of pathogen exposure probability of different species (Supplementary Annex). This considered aspects such as contact with infected or diseased animals, contact to wild birds, species-specific vulnerability to HPAI, and availability of indoor enclosures to keep birds for an extended time period. In addition, available care and husbandry options by animal keepers had to be scrutinized as well as indoor housing tolerance, and options for single-species and mixed-species group housing (socialization capability) of different bird species (Figure 2).

Extended hygiene procedures, in addition to the high standards of a confined establishment, aimed to avoid exposure of animal keepers and veterinarians so as to reduce the risk of them becoming indirect vectors between epUs inside the zoo or outside. Hygiene locks were installed at the entrance of all epUs. Personal protective equipment, consisting of disposable coverall and gloves, boot covers and FFP3 masks had to be worn during all sampling, feeding, cleaning and disinfection operations. The sampling scheme as detailed in paragraph 3.1.3, comprising the order of sampling, sampling material and sampling intervals, was determined and specified by the competent authority counseled by CVUA. Sampling was supervised by official veterinarians.

### 3.2 Restricted spread of HPAIV in the zoo

#### 3.2.1 Clinical and virological monitoring revealed mild courses of infection

Following outbreak confirmation (Day 0) serial sampling and clinical scoring was initiated to monitor the putative spread of HPAIV within and between the different epUs. Clinical signs that were observed included apathy, inappetence and neurological disorders such as nystagmus and circular movements. Eleven infected animals of four species died and 15 infected individuals of four species were euthanized. A further 7 non-infected birds died (including four by euthanasia) during the outbreak.

In total, 3,634 swab samples were examined by RT-qPCR during a 74 days period. At Day +1 after the outbreak confirmation an initial round of sampling (combined choanal and cloacal swab sample collection) was performed for individuals at high risk (*n* = 87) in five epUs (no. 10–12, 18 and 19; Figures 1, 2), containing birds that had access to the pond areas. About one third of the swabs (*n* = 28) were declared positive with higher likelihood of representing increased infectivity (ct < 30). Viral loads of about two thirds of those swabs (*n* = 52) remained positive with less likelihood of representing increased infectivity (*n* = 52; 30 < ct < 40), while seven individuals tested negative (ct ≥ 40). RT-qPCR positive individuals were restricted to epU 18, 19 and 10.

**Figure 4:**
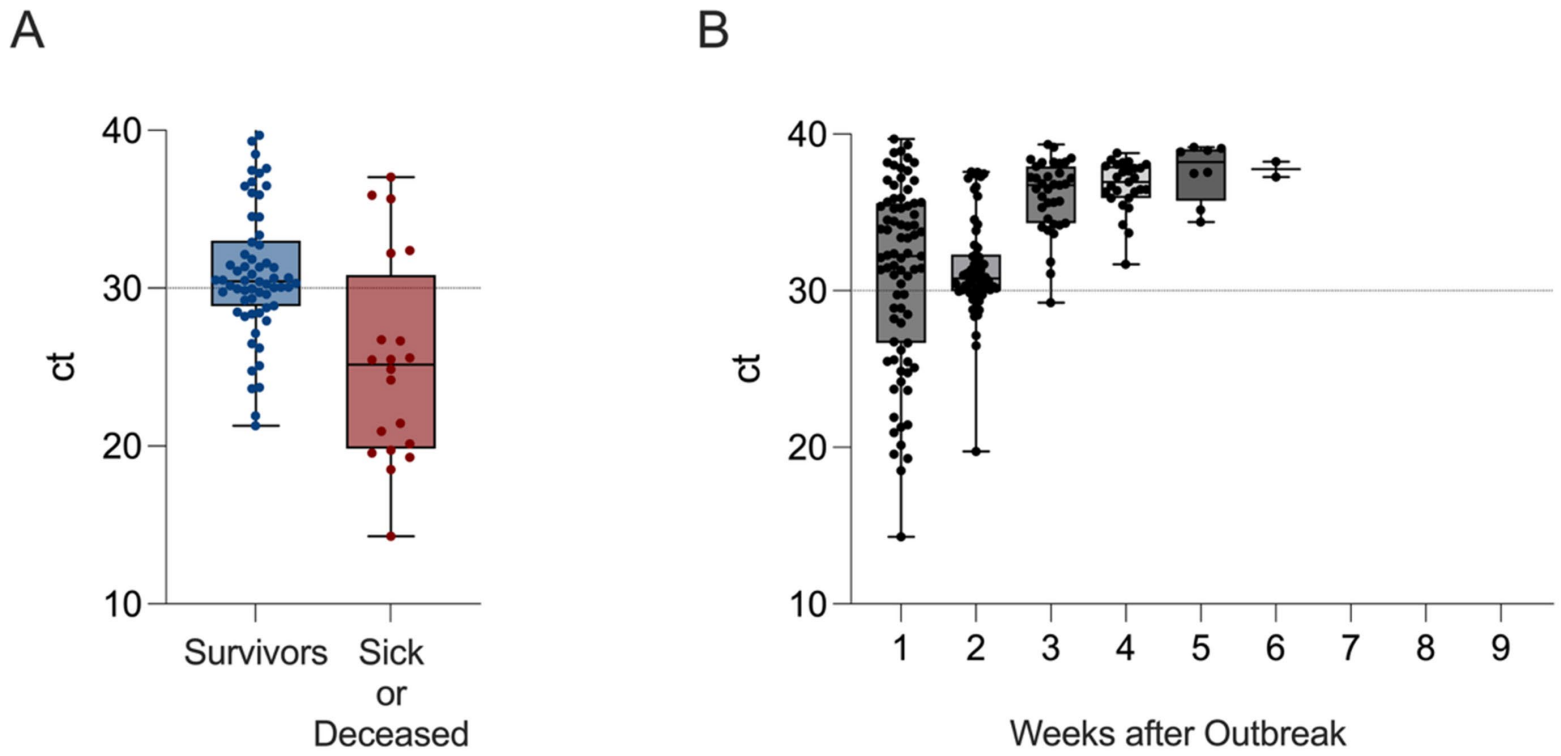
Comparison of maximum virus loads measured by RT-qPCR linked to **(A)** clinical outcome in individual birds (blue – survivors without clinical signs of avian flu, red – severe clinical signs and/or fatal outcome), and **(B)** timeline of all RT-qPCR-positive zoo birds (black) after incursion of HPAIV H5N1 into the Zoological Garden Karlsruhe, Karlsruhe, Germany. Cycle of threshold values (ct) are depicted as box plots with single data points, minimum and maximum range. The dashed line represents an arbitrary ct distinguishing viral loads that were deemed to be associated with a higher (ct ≤ 30) or lower likelihood (30 < ct < 40) of efficacious virus transmission. Individuals revealing ct values of ≤30 in the initial swab sample tend to characterize birds that developed or expressed clinical signs or deceased in the course of the HPAI outbreak **(A)**. The overall highest viral loads had been detected during the first 2 weeks of sampling **(B)**, while viral genome could be detected until week 6 after the HPAI outbreak.

Mortality (including euthanasia) occurred only within the three heavily affected epUs (epU18 [7 Hawaiian geese and 1 Cape Barren goose (*Cereopsis novaehollandiae*)], epU19 [1 *Branta leucopsis* and 1 *Anser cygnoides*] and epU10 [10 *Pelecanus onocrotalus*]) and was limited until Day +8. In total, within the first 2 weeks of the outbreak about one quarter of the initially RT-qPCR positive individuals succumbed to the disease or had to be euthanized (21/79 individuals, 26.6%, ct range 14.3–39.7), while the remaining individuals showed no clinical signs despite being RT-qPCR positive. There was a tendency toward a fatal course of the infection in birds with higher viral loads (ct < 30; Figure 4A). From week 3 onwards only swab samples from epUs 19 and 10 turned out to be IAV-positive, lasting until week 4 and 5 with constantly decreasing virus load until swab samples of all epUs were negative (Figure 4B). Overall, the first animals that tested RT-qPCR positive returned to negative status in week 3.

No mammal species tested in the zoo were found positive by RT-qPCR; no samples were obtained for serological analyses. During the entire outbreak investigation none of the staff members reported signs of an influenza-like illness. Regarding the limited zoonotic propensity of the associated HPAIV strain (14), no accompanying virological or serological surveillance of staff was deemed justified by the competent authorities, but could of course be considered in other cases.

#### 3.2.2 Whole genome sequencing elucidated HPAIV H5 incursion and spreading patterns in the zoo

Eight wild bird samples from the Karlsruhe region have been applied to whole-genome sequencing originating from a Canada goose (*n* = 1; *Branta canadensis*), swans (*n* = 2), and gray herons (*n* = 5). This comprised the gray heron index case from January 2022 (Supplementary Table S1) and four further gray herons, with suspected contacts to the zoo area. Complete coding sequences (CDS) were gained for all IAV genome segments of these eight wild bird samples and confirmed the RT-qPCR results of two different subtypes, H5N1 and H5N2, and their HP pathotype. Both H5N2 samples were assigned to genotype Eurasian (EA) AU, whereas the subtype H5N1 samples represented two distinct genotypes, EA AB (*n* = 5) and EA C (*n* = 1, Canada goose). All genotypes share the same HA and M gene present in all current gs/GD-like HPAIV H5 subtypes in Europe. Also genotypes EA C, EA AB and EA AU contain the same polymerase basic 1 segment. Although three distinct genotypes were present in wild birds in the region during the time of zoo incident, only one of these genotypes (EA AB) was detected in the zoo samples. This finding underlines the strength of molecular data for mapping possible routes of incursion from infected wild birds into the zoo.

In addition, eleven zoo bird samples were sequenced including samples from Great white pelicans (*n* = 4), Muscovy ducks (*n* = 2; *Cairina moschata*), Barnacle goose (*n* = 1; *Branta leucopsis*) and Hawaiian geese (*n* = 4). All samples were obtained at the onset of the outbreak (Figure 2). All genomes were assigned to genotype EA AB. All CDS shared sequence identity levels of 99.89–100% and revealed an overall close phylogenetic clustering (Supplementary Figure S1). Median joining network analysis showed that the sequences of the wild bird (gray heron) index cases take a central position in the network with all sequences of zoo birds being localized at more peripheral sites (Figure 3). This strengthened the suspicion of an incursion from wild birds, likely including gray herons, visiting the ponds at the zoo premises.

#### 3.2.3 Serological investigations suggest evidence for the development of protective and long-term immune responses

Prior to the outbreak, blood samples in the zoo had been collected occasionally in the context of routine health screenings, transport examinations and *ad hoc* veterinary diagnostic procedures (*n* = 21; 10 taxonomic orders; Anseriformes, Ciconiiformes, Columbiformes, Gruiformes, Phoenicopteriformes, Psittaciformes, Rheiformes, Sphenisciformes, Strigiformes and Struthioniformes). Only three pre-2022 sera (all originating from Chilean flamingoes [*Phoenicopterus chilensis*]) tested positive for IAV antibodies (14.3%), but none of those samples proved to be positive for antibodies against subtype H5.

**Figure 5:**
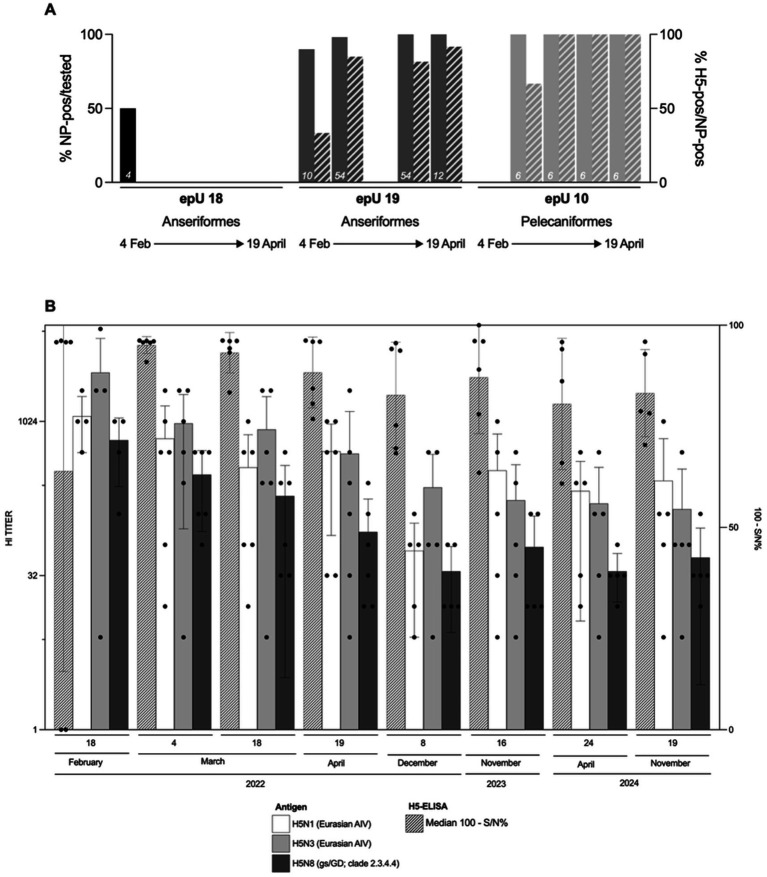
Seroconversion of zoo birds in affected epidemiological units (epUs) during the outbreak phase **(A)** and long-term immune response surveillance in a cohort of six pelicans affected during the outbreak phase until 2.5 years after the outbreak **(B)**; one pelican left the zoo in the beginning of 2024. While no individual bird in epU 18 could be tested after the first round of sampling, individuals in epU 19 and epU 10 showed increasing seroconversion specific for subtype H5 within 2.5 months after the onset of the outbreak **(A)**. The group of pelicans showed a similar long-term immune response in form of H5-specific antibodies (H5-ELISA results), including those specific for HPAIV H5 of clade 2.3.4.4 (HI results) up to 2.5 years after the outbreak.

During the outbreak phase, between early February and mid-April 2022, a total of 196 serum samples had been collected from zoo birds of six different taxonomic orders (Anseriformes, Gruiformes, Pelecaniformes, Psittaciformes, Rheiformes and Sphenisciformes) (Figure 5A). Repeated sampling was possible from six zoo-housed Pelecaniformes. Some epUs with RT-qPCR-positive birds (epUs 10, 18 and 19) revealed H5-seropositive birds during the initial and all subsequent rounds of blood sampling (49/64 samples in week 3, 6/6 in week 5, 50/60 in week 7 and 18/37 in week 9 after the outbreak). Notably, antibodies against subtype H5 were detected in a single individual (Greater scaup [*Aythya marila*], *n* = 1) of epU 14 in week 11 after the outbreak without being tested serologically nor being tested positive by RT-qPCR before (Figure 2). There was no indication for a recent infection in this bird or the associated epU. However, as no previous samples of this adult bird exist for comparison, a prior contact to any AIV of subtype H5 and an anamnestic reaction upon a recent antigen contact could be a plausible explanation for seroconversion. In other RT-qPCR-negative epUs (1, 4, 12, 22 and 25), no H5-specific seroconversion was detected.

H5-seroconversion based on repeated sampling of individual birds was exclusively detected in Pelecaniformes and Anseriformes species. The outbreak-related sample set showed an initial increase of H5-seroprevalence in February.

After cessation of the outbreak in April 2022, further 124 serum samples had been collected until mid-November 2024 in the context of routine health screenings, transport examinations and *ad hoc* veterinary diagnostic procedures from 12 taxonomic orders (Anseriformes, Bucerotiformes, Ciconiiformes, Galliformes, Gruiformes, Pelecaniformes, Phoenicopteriformes, Piciformes, Psittaciformes, Rheiformes, Sphenisciformes, and Strigiformes). Of 47 samples with NP-specific antibodies (47/124, 37.9%), 27 samples also tested H5-positive (Anseriformes, *n* = 3; Pelecaniformes, *n* = 22, and Phoenicopteriformes, *n* = 2). Great white pelicans were the most regularly sampled birds during the outbreak investigations (epU 10) and afterwards. Figure 5B illustrates serological results of the pelican samples at eight time points. Birds remained H5-seropositive by ELISA and HI showing fairly stable titers over at least 2 years. While three different H5 antigens were employed in HI assays, the 2.3.4.4 antigen did not yield the highest titers in comparison to two Eurasian low pathogenicity H5 antigens.

## 4 Discussion

The HPAI H5 outbreak in the Zoological Garden Karlsruhe took place in early 2022 within the first peak of an unprecedented massive circulation of gs/GD-like HPAIV H5 in wild birds in Europe. Within 6 months, between January and July 2022, Germany notified almost one thousand cases in wild birds and more than 30 outbreaks in captive birds, including flocks in two different zoos (15). Due to the potentially devastating impact in case of the spread of HPAI H5 in domestic and wild birds and its zoonotic potential with possible public health implications, the European animal health legislation basically requires the culling of all captive birds in affected holdings as an attempt to arrest lateral virus spread between holdings and spill back into wild bird populations. If unwittingly applied to zoological institutions, such operations could result in the death of thousands of zoo birds, including highly protected, endangered species and individuals of high genetic, cultural or educational value, many of them being managed in international *ex-situ* conservation programs (e.g., EAZA *ex-situ* program, EEP) aiming to maximize species conservation and biodiversity. Considering that, zoos can request exemptions from culling by their competent veterinary authority. In contrast to previous outbreaks in zoos (4) the competent veterinary authorities handling the Karlsruhe case presented here granted permission for infected, but healthy, animals to recover and seroconvert. This required that (i) all avian species were segregated into isolated epUs, (ii) HPAI infections remained confined to the initially affected epUs (as testified by extensive RT-qPCR investigations), (iii) frequent clinical monitoring allowed to identify animals with clinical signs, (iv) clinically diseased birds approaching pre-defined humane endpoints were euthanized, and (v) precautions were taken to prevent spill-over infection to personnel and public.

Based on the molecular and phylogenetic data, the initial virus introduction, likely from local infected wild birds, might have happened late January/early February 2022, rather days than weeks before the zoo bird index case was confirmed on February 3, 2022. These data also demonstrate the importance of early detection and testing for HPAIV H5. It is crucial to conduct active surveillance and testing at the earliest possible stage. While the sequencing of HPAIV H5 in wild birds revealed three different genotypes circulating back then, only genetically very similar sequences of HPAIV subtype H5N1 of genotype EA AB, clade 2.3.4.4b, were detected in affected zoo birds and in wild birds roaming at the same time in the region and directly in the zoo (gray heron colony). All individuals of the initially affected epUs 10, 18 and 19 had been kept in close proximity to each other on the zoo’s ponds. Initial exposure of zoo birds with access to this fresh water source and thus to possibly infected wild birds therefore emerges as the most likely scenario of virus incursion in form of a single genotype and initial spread.

Epidemiological observations (e.g., clinical signs and mortality) of the course of the outbreak are consistent with subsequent laboratory analyses and suggest the successful containment of the pathogen within the initially affected units (Figure 4). The decreasing detection of viral genome RNA in frequency and load over time was associated with increasing H5-specific seroconversion rates and HI antibody titers. Lack of seroconversion in other units also proved the restriction measures successful in excluding HPAIV spread.

Individual birds showing clinical signs, including apathy, inappetence and/or neurological disorders inevitably turned out to be infected. In the cohort of all infected individuals (*n* = 79) mortality reached a level of about one quarter. Infection-associated deaths were restricted to the first 8 days. Progression to a fatal course of disease was associated with higher virus load in swab samples (Figure 4B). This elucidates the need for prompt and rigorous initial measures of containment (Figure 4A). Spill-over between units was blocked efficiently, although measures had been applied throughout all avian holdings within the zoo and no control group can serve as comparison for this claim. Strict indoor housing orders, however, associated with higher population density and negative effects on animal welfare, may have enhanced virus spread within units already harboring infected individuals. This may have led to increased infection-associated mortality during housing. In addition, seven uninfected birds died during the indoor housing period. These cases may have been promoted by increasing stress levels due to the implementation of the isolation measures.

Exclusively, anseriform and pelecaniform individuals had been detected RT-qPCR positive and some individuals of both groups developed clinical signs, deceased or had to be euthanized. However, not all RT-qPCR positive birds developed clinical signs. Asymptomatic courses of disease were associated with lower loads of viral RNA in swab samples (Figure 4B). This justifies the decision of the competent authority not to cull all infected birds. It also highlights the inevitable necessity of extensive surveillance via sampling and virological investigations to assess the entire dimension and dynamics of an outbreak in a zoo.

The surveillance data show that any epU containing infected animals was unlikely to return to a fully negative PCR status before a period of 3–4 weeks. Therefore, in a similar scenario, increased sampling frequency during the first 3 weeks after housing will not be helpful in speeding up clearance of the zoo. On the contrary, circumstances of repeated catching and sampling provides increased opportunities for spill-over or contamination with HPAIV H5Nx and might negatively affect the birds’ capacities to cope with the infection and housing stress. Therefore, a second sampling round could be postponed until at least 3 weeks after the first one, offering a reasonable compromise between animal welfare and animal disease management.

In conclusion, success in containing an HPAI H5 outbreak in the Zoological Garden Karlsruhe with a large avian population was based on several factors including

- A balanced implementation of current legislation, in particular to link culling to the development of clinical signs and not to mere detection of viral RNA.
- Pre-outbreak preparations of the zoo.

◦ Plan ahead with the creation of epUs that could be assembled rapidly (“*Segregate*”).
◦ Create serum banks to prove freedom from a particular infection prior to outbreaks and for comparative analyses.

- Rapid action for early detection in case of any suspicion for HPAIV and other notifiable infections (e.g., West Nile virus) (“*Test*”).
- Application of adaptable measures during the quarantine period (“*Observe*”).
- Compliance with strict hygiene requirements.
- Perseverance of the zoo in tolerating closing and housing orders (“*Persevere*”).

For reasons of animal welfare and animal disease control, it was not possible in this case to establish a control group in order to quantify or compare the effectiveness of the measures.

Similarly, it is impossible to draw recommendations regarding the “*STOP*” strategy for zoos in general, as every zoo is different with highly variable and diverse structures to rear captive birds in comparison to the rather uniform layout of poultry holdings. In addition, factors such as the type of HPAIV strain, the range of avian species, their health status etc. will vary considerably, rendering each outbreak a unique challenge. Vaccination of zoo birds against HPAIV may add an important preventive measure. Efficacious vaccines against the 2.3.4.4b lineage of HPAIV H5 expected to be safe also in use with the diverse species range of zoo birds are becoming available (16). While carefully selected vaccines and adapted vaccination programs may offer advantages in adapting control measures by competent authorities, it may also come with restrictions in other aspects, e.g., movement restrictions of vaccinated birds threatening an international exchange of vaccinated birds in *ex-situ* species conservation and breeding programs. In this regard, the use of vaccinations necessitates meticulous consideration, encompassing a thorough evaluation of the epidemiological landscape and the particular risk of introduction into the zoo, juxtaposed with the potential constraints on the unrestricted application of birds in breeding programs.

## Data Availability

The datasets presented in this study can be found in online repositories. The names of the repository/repositories and accession number(s) can be found in the article/Supplementary material.
