# Supplementary material for: Segregate, Test, Observe and Persevere (STOP): strengthening ex situ breeding programs for biodiversity in zoos amid highly pathogenic avian influenza threats - a case approach
Source: Front Vet Sci. 2026 Mar 6;13:1708354. doi: 10.3389/fvets.2026.1708354 (PMC13002437; doi:10.3389/fvets.2026.1708354)
Supplement: Supplementary file 3 [file Supplementary_file_1.docx]

**Supplemental Annex**

**Manuscript: 'Segregate, Test, Observe and Persevere' (STOP): Strengthening ex situ breeding programmes for biodiversity in zoos amid highly pathogenic avian influenza threats – a case approach**

*Anne Günther^1^, Marco Roller^2^, Lukas Reese^2^, Ulrike Foldenauer^3^, Judith Tyczka^4^, Anne Pohlmann^1^, Martin Beer^1^, Dominik Fischer^5^, Timm Harder^1^*

#### Legal framework

Following the official confirmation of an outbreak of a category A disease in an establishment in the EU, the competent authority shall order that specific disease control measures are immediately applied under the supervision of official veterinarians. According to Article 12 (1) (a) of Commission Delegated Regulation (EU) 2020/687 all animals of listed species kept in the affected establishment shall be killed as soon as possible on the spot, within the establishment, in such a way as to avoid any risk of spreading the relevant category A disease agent during and after killing. The competent authority may grant derogation from Article 12 (1) (a) provided that animals are kept in a confined establishment, kept for scientific purposes or purposes related to conservation of protected or endangered species, are officially registered in advance as rare breeds and for animals with a duly justified high genetic, cultural or educational value (Article 13 (2) (a-d) Commission Delegated Regulation (EU) 2020/687).

The reopening concept comprised complete restriction of visitors access until 7th March when the zoo was reopened partially. Still, biosecurity measures aimed at avoiding inadvertent spread of virus through contaminated fomites were continued. For the final revocation of measures the following conditions were predetermined in accordance with Commission Decision 2006/437/EC: (i) all formerly infected animals tested negative; (ii) buildings and equipment potentially contaminated had been cleaned and sanitized under official control; (iii) epUs were tested twice with negative results at day 21 and 42 after the last virus detection. These conditions were fulfilled on 20th April 2022 and revocation of all control measures was granted.

#### Summary of “Genotype differentiation of highly pathogenic avian influenza viruses (HPAIV) of the goose/Guangdong lineage in Germany - Derivation and deployment of reference sequences.” by Pohlmann and Harder (1)

For the full text and all references refer to: <https://zenodo.org/records/17533574>

Genotypes and reference sequences were determined using a combined phylogenetic and similarity-based approach. Here, HPAIV H5 positive samples have been retrospectively included in the continuous screening for new genotypes and compared with derived references. Segment-specific and concatenated sequence alignments were generated with MAFFT (Katoh & Standley, 2013), followed by the calculation of maximum-likelihood trees for each segment and the concatenated genome in RAxML (GTR-GAMMA, rapid bootstrapping, 1000 replicates; Stamatakis, 2014). Genotypes were assigned—and new ones defined—when sequences formed well-supported monophyletic clusters and showed differences in normalized patristic distances at the segment level.

#### Reference

1. Pohlmann A, Harder T. Genotype differentiation of highly pathogenic avian influenza viruses (HPAIV) of the goose/Guangdong lineage in Germany - Derivation and deployment of reference sequences Zenodo2023 [cited 2025 14.08.2025]. Available from: <https://doi.org/10.5281/zenodo.8233814>.
