# Supplementary material for: Segregate, Test, Observe and Persevere (STOP): strengthening ex situ breeding programs for biodiversity in zoos amid highly pathogenic avian influenza threats - a case approach
Source: Front Vet Sci. 2026 Mar 6;13:1708354. doi: 10.3389/fvets.2026.1708354 (PMC13002437; doi:10.3389/fvets.2026.1708354)
Supplement: Supplementary file 2 [file Image_1.pdf]

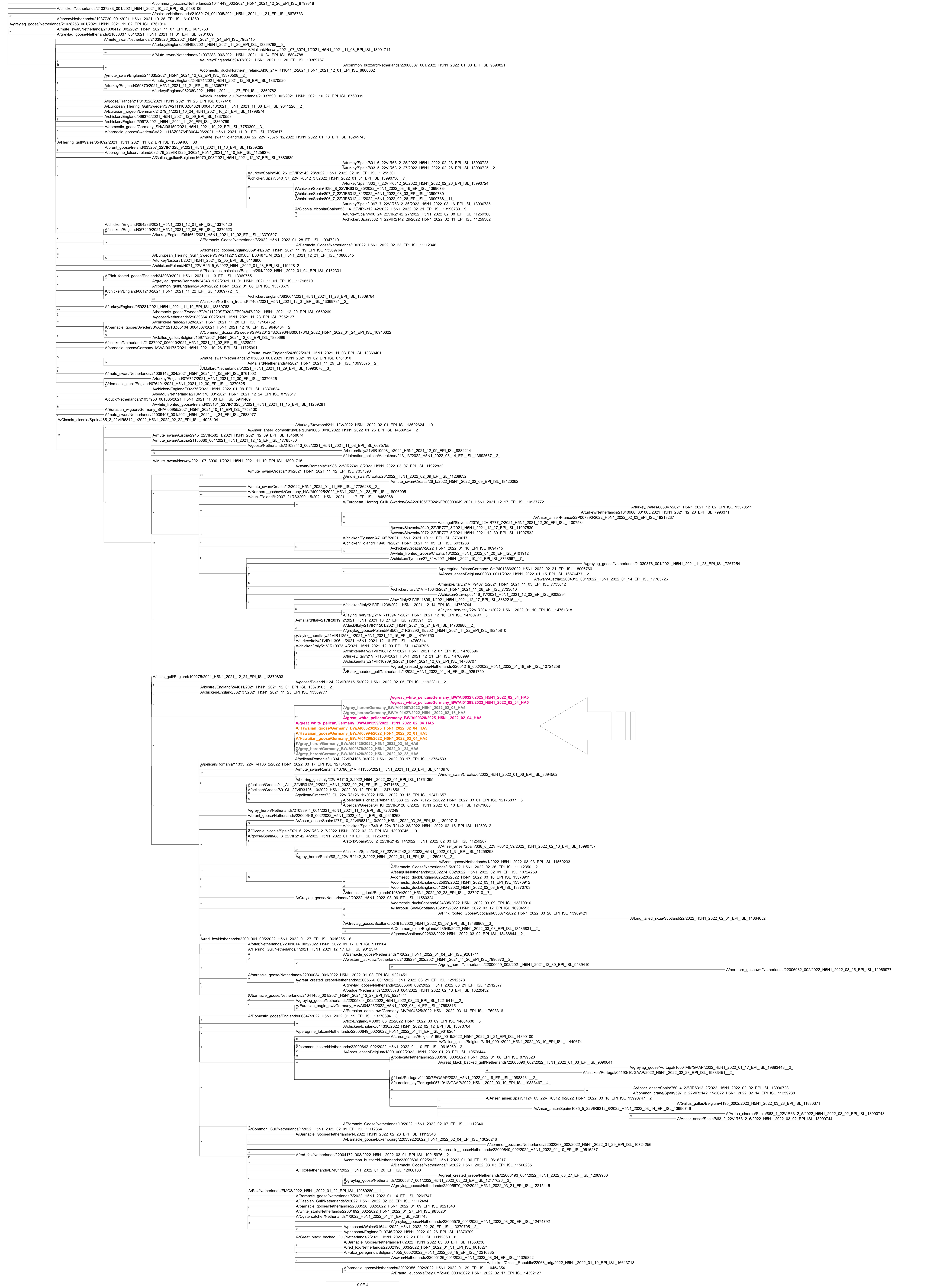
9.0E-4
